# A method based on interpretable machine learning for recognizing the intensity of human engagement intention

**DOI:** 10.1038/s41598-023-29661-2

**Published:** 2023-02-13

**Authors:** Jian Bi, Fang-chao Hu, Yu-jin Wang, Ming-nan Luo, Miao He

**Affiliations:** College of Mechanical Engineering, Chongqing University of Technology, Chongqing, China

**Keywords:** Mechanical engineering, Computational science, Computer science

## Abstract

To interact with humans more precisely and naturally, social robots need to “perceive” human engagement intention, especially need to recognize the main interaction person in multi-person interaction scenarios. By analyzing the intensity of human engagement intention (IHEI), social robots can distinguish the intention of different persons. Most existing research in this field mainly focus on analyzing whether a person has the intention to interact with the robot while lack of analysis of IHEI. In this regard, this paper proposes an approach for recognizing the engagement intention intensity. Four categories of visual features, including line of sight, head pose, distance and expression of human, are captured, and a CatBoost-based machine learning model is applied to train an optimal classifier for predicting the IHEI on the dataset. The experimental results show that this classifier can effectively predict the IHEI that can be applied into real human–robot interaction scenarios. Moreover, the proposed model is an interpretable machine learning model, where interpretability analysis on the trained classifier has been done to explore the deep associations between input features and engagement intention, thereby providing robust and effective robot social decision-making.

## Introduction

Social robots have become more and more popular in society and this trend has created many new opportunities for various industries^1–3^. Among the techniques used in social robots, recognition of the intention of a user to engage in an interaction with a robot can improve the proactivity of social robot interaction in a more natural way. Although robots can recognize human intentions of engagement (HIE) by understanding verbal instructions, in such cases, humans have to give very clear commands to robots while robots can only respond passively, which does not really make sense for what social robots are supposed to be designed for. In addition, for those social robots with human-like appearances, such as Nadine^4^, without natural interactions, they might scare other people.

**Figure 1:**
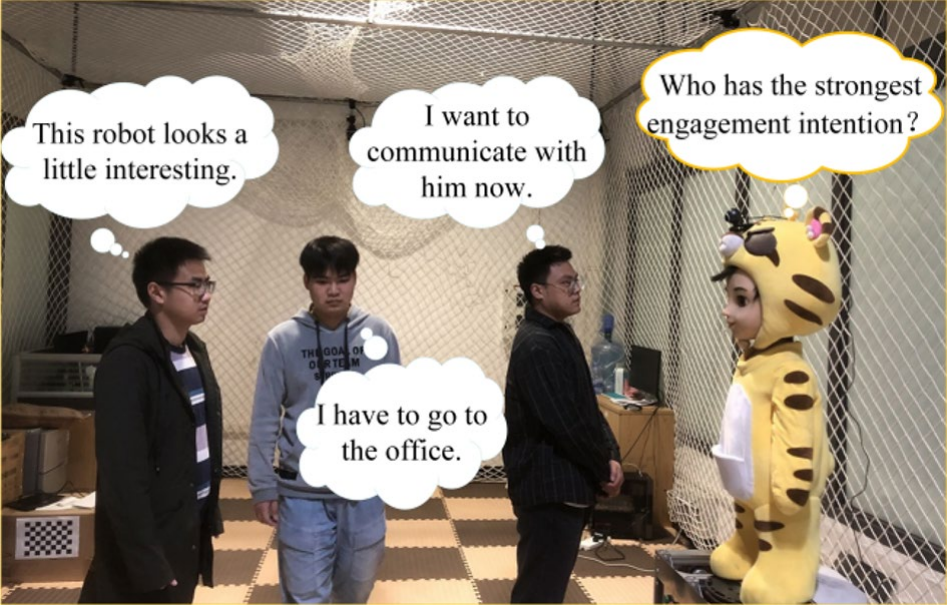
A scene where a social robot interacts with multiple people. These three people are each thinking, “This robot looks a little interesting” (left), “I have to go to the office” (middle), and “I want to communicate with him now” (right).

To improve the naturalness of these social robots, we need to improve not only the fluency of their actions, but also their perception capabilities for social environments to enhance the initiative of interactions. HIE recognition facilitates quick decision-making in an interactive agent, and makes social robots respond to humans’ needs or psychological states in a better way. Therefore, it can improve the naturalness and harmony of human–robot interaction (HRI). For instance, in the scene shown in Fig. 1, there are three people with different interactive intentions in front of the social robot. The social robot needs to distinguish the engagement intentions of the three people in the picture in order to reasonably select objects for more natural interaction. As for HIE recognition, the existing methods of HIE recognition can be divided into two types: social rule-based and data-driven methods.

The HIE recognition methods based on social rules apply psychological and sociological theories or related experimental results to establish discriminant criteria and models to identify HIE, while distance is an important factor of social intentions. Many previous studies^5–8^ used Hall’s proxemics^9^ to model HIE recognition. And Walterset al.^10,11^ studied more deeply on human–robot proxemics. Furthermore, gaze^12,13^, head position^14^, gesture^15^, motion trajectory^16,17^ and other features have been widely used for HIE recognition. However, the above methods require professional knowledge of social science and effective social experimental results. Moreover, the validity of the results might depend on the experience of each researcher. Therefore, complex rules are often necessary to guarantee the validity of HIE recognition.

With the advantage of artificial intelligence technology and the improvement of computer processing performance, the data-driven HIE recognition method has been adopted by more and more researchers. Yusuke et al.^18^ used the people tracking system (3-D Range Sensors) in a shopping mall to collect a total of 130 pedestrians’ movement data, and each person was recorded for 10 s on average. The data information included distance, deflection angle, speed and time being stopped of a pedestrian. Then, they used support vector machines (SVM)^19^ to identify whether pedestrians are willing to interact with the robot. Vaufreydaz et al.^20^ chose a home-like environment as the interaction scene. Fifteen participants were arranged to enter the room with the robot in succession and choose whether to interact with it. The sensor system on the robot extracted the spatial features of participants and finally obtained 158,200 frames of data information. They used SVM and neural networks to train the dataset respectively, and found that the test effect was best when the interaction state was divided into WILL_INTERACT, NO_ONE and SOMEONE_AROUND. Sidiropoulos et al.^21^ studied the level of engagement of children in the interaction with social robots in special education. They selected 10 children aged 9–10 for the experiment, and extracted their facial, body and voice features to obtain 2497 samples, of which each sample had 60 s. Different from other studies, this study treated the estimate of participation as a regression problem, with a label value of thought set and a value range of 0–1. Then they used SVM, Linear Regression (LR)^22^, Multi-layer Perceptron (MLP)^23^ and Random Forest (RF)^24^ to train the regression model respectively, and finally found that MLP had the best effect. However, the determination of engagement level in this paper is too subjective.

The data-driven HIE method does not rely on complex internal mechanisms, and it is not necessary to establish specific mathematical models and interaction rules. This type of approach can usually achieve high recognition accuracy and can be flexibly updated. Nevertheless, the above-mentioned studies only assess whether an individual has an intention to interact with robots, without analyzing the intensity of human engagement intention. To address this challenge, this paper proposes an interpretable machine learning model based IHEI recognition method to explore the correlation between the IHEI and visual features.

## Method

**Figure 2:**
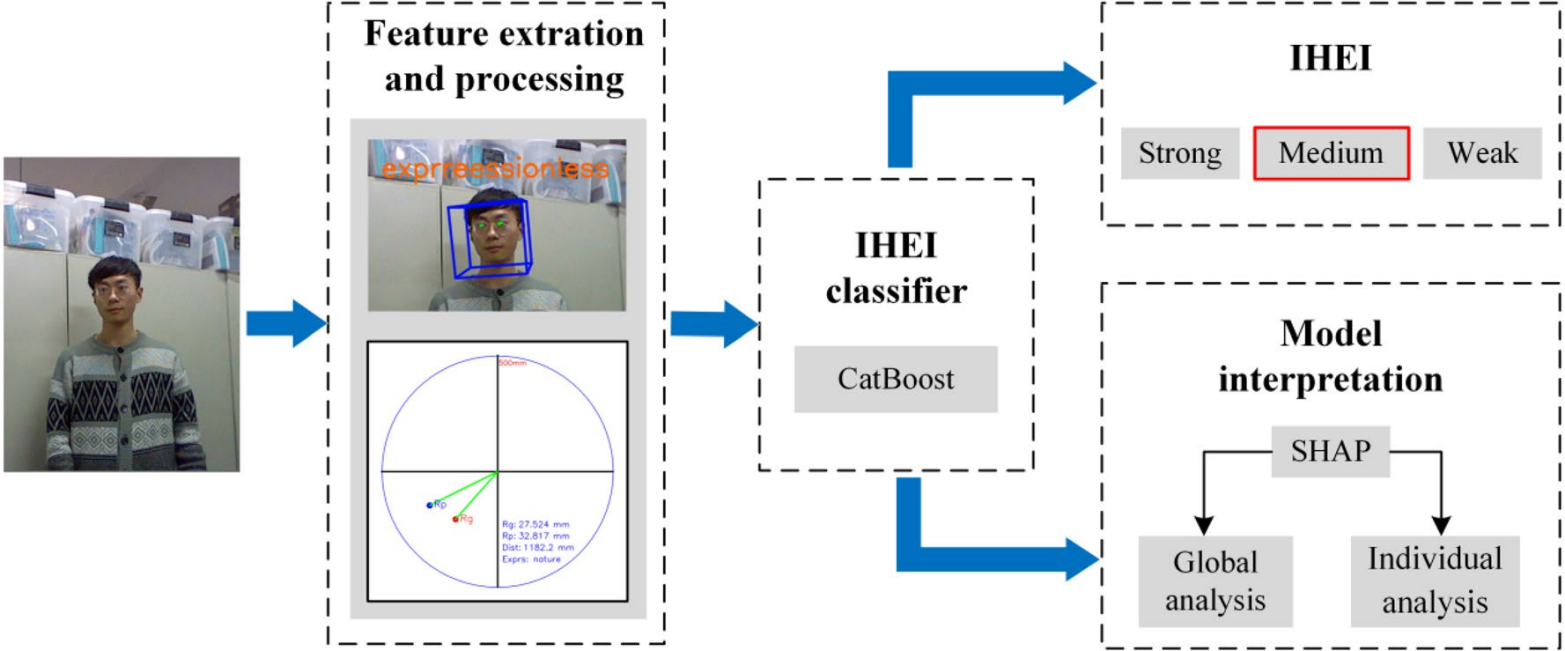
Flow chart of our method.

In this paper, the intensity recognition of HIE is classified based on CatBoost^25,26^ and model interpretability is analyzed using SHAP^27^. The flow chart of our proposed method is described in Fig. 2 below. First, we use OpenFace2.0 toolkit^28^, which is a face analysis tool based on in-depth learning, and a feature processing tool to get the visual feature data of line of sight, head pose, distance and facial expression. Then, we regard the IHEI recognition as a multi-classification problem, and classify the IHEI into three categories: Strong, Medium and Weak, which can be distinguished according to respective intensity values. Different intensity values of HIE are used as label sets, and the visual feature data we extract is used as feature sets. We use CatBoost as the classifier, and then conduct an interpretable analysis of the trained classifier and use SHAP to explore the deep correlations between feature sets and IHEI.

All studies reported in this paper were approved by the Human Ethical Committee at Chongqing University of Technology. All methods were performed in accordance with the relevant guidelines and regulations. All participants gave written informed consent before the experiment. After the experiment, all participants were debriefed about the purpose of the study.

### Feature extraction

Our experiment makes use of the features of human line of sight, head pose, distance, and facial expressions, as these are visual attributes that correlate strongly with the potential for HIE. These features can be extracted simultaneously by only one visual sensor which is very convenient for our follow-up work. The existing deep learning library can extract these features effectively and ensure the validity of subsequent experiments. Specifically, we extract these features with OpenFace2.0 toolkit through a calibrated monocular camera.

#### Line of sight

Line of sight provides powerful clues to people’s intentions, motivations, and attention. OpenFace2.0 toolkit uses a feature detector based on Constrained Local Nerve Field (CLNF)^29,30^ to detect the iris and pupil, and obtain the coordinates of each landmark in the 3D camera coordinate system to calculate the line of sight direction. Using the OpenFace2.0 toolkit, we can obtain the direction vector data **{*v***_***glx***_*, ****v***_***gly***_*, ****v***_***glz***_}, **{*v***_***grx***_*, ****v***_***gry***_*, ****v***_***grz***_**}** and eye line azimuth data {***G***_***yaw***_*, ****G***_***pitch***_} of the binocular line of sight of human.

#### Head pose

**Figure 3:**
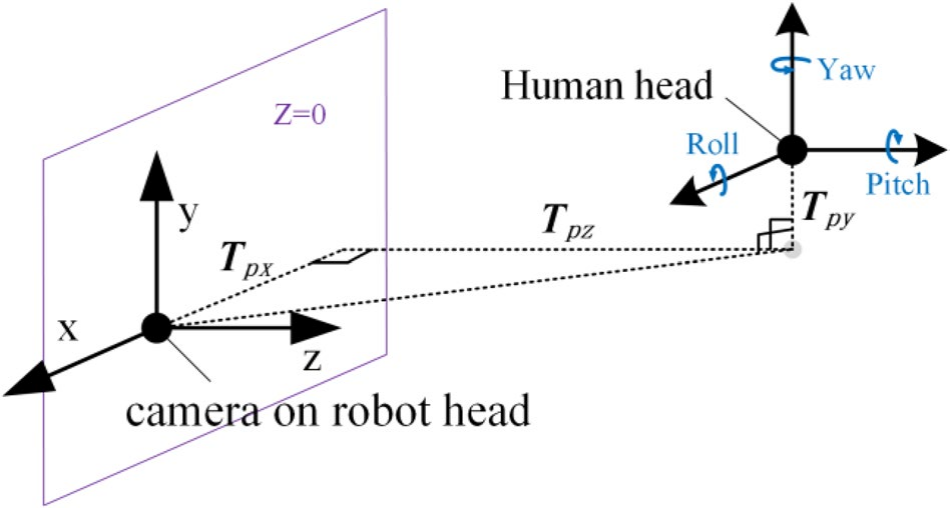
Head pose estimation in a camera coordinate system.

As for the feature of head pose, facial orientation can indicate the direction in which the person is most interested at the moment. For head pose estimation, OpenFace2.0 uses the Convolutional Experts Constrained Local Model (CE-CLM)^31^ to obtain the 3D representation of facial landmarks. Then it uses orthogonal camera projection to project the facial landmark to the image. Finally, the translation change {***T***_***px***_, ***T***_***py***_, ***T***_***pz***_} and the rotation change {***P***_***pitch***_, ***P***_***yaw***_, ***P***_***roll***_} of the head pose can be obtained by the perspective transformation. The head pose representation in the camera coordinate system is shown in Fig. 3.

#### Distance

The translation change of head pose can reflect the distance change of a human relative to the robot. According to the feature set from the Section of head pose, it can be seen that the 3D coordinates of the center of the human head (face center point) from the perspective of the robot are {***T***_***px***_, ***T***_***py***_, ***T***_***pz***_}, as shown in Fig. 3, where ***T***_***px***_ reflects the horizontal left and right movement of human head relative to the robot, ***T***_***py***_ reflects the vertical movement change, and ***T***_***pz***_ reflects the horizontal depth movement change.

#### Facial expressions

Human beings intuitively convey information about their emotions and state of mind through their facial expressions^32,33^. Especially, facial expression recognition plays an important role in emotional interaction. We divide facial expressions into six basic categories: happy, sad, surprised, scared, angry, and disgusted.

In order to effectively identify the six common facial expressions and obtain our feature data ***E***, we use OpenFace2.0 to extract the intensity and existence of Action Unit (AU) feature in the Facial Action Coding System (FACS)^34^. We then distinguish human facial expressions through the correspondence between different expressions and AUs^35^ as shown in Table 1.

**Table 1:** The correspondence between basic expressions and AUs.

***E***	AUs
Happy	6 + 12 + 26
Sad	1 + 4 + 15
Surprised	1 + 2 + 5 + 26
Scared	1 + 2 + 4 + 5 + 7 + 20 + 25,26
Angry	4 + 5 + 10 + 25
Disgusted	4 + 9 + 17

### Feature engineering

#### Enhance readability of feature data

From the previous section, we can get 15 types of feature data related to line of sight, head pose, distance, and facial expressions. However, the readability of the feature data is not clear and cannot intuitively reflect the direct correlation between each feature and HIE. Therefore, the feature data need to be processed to improve the correlation between each feature and HIE and enhance readability.

In order to intuitively read the line of sight feature and enable our model to train this feature better, we reduce the dimension of this feature to obtain the one-dimensional attention shift coefficient ***R***_***g***_. ***R***_***g***_ is obtained by calculating the offset distance between the falling point of the human line of sight and the center point of the robot face. To simplify the analysis, we assume that the line of sight direction of human eyes is the same. The line of sight landing point is the average landing points of left and right eyes. Therefore, the calculation formula for ***R***_***g***_ is as follows:

1 $$\begin{gathered} \hfill {\varvec{R}}_{{\varvec{g}}} = \left( {\left( {\frac{1}{2}(p_{xl} - p_{zl} \frac{{{\varvec{V}}_{{{\varvec{glx}}}} }}{{{\varvec{V}}_{{{\varvec{glz}}}} }} + p_{xr} - p_{zr} \frac{{{\varvec{V}}_{{{\varvec{grx}}}} }}{{{\varvec{V}}_{{{\varvec{grz}}}} }})} \right)^{2} } \right. + \\ \hfill \left. {\left( {\frac{1}{2}(p_{yl} - p_{yl} \frac{{{\varvec{V}}_{{{\varvec{gly}}}} }}{{{\varvec{V}}_{{{\varvec{glz}}}} }} + p_{yr} - p_{zr} \frac{{{\varvec{V}}_{{{\varvec{gry}}}} }}{{{\varvec{V}}_{{{\varvec{grz}}}} }})} \right)^{2} } \right)^{\frac{1}{2}} \\ \end{gathered}$$

where [*p*_*xl*_*, p*_*yl*_*, p*_*zl*_] and [*p*_*xr*_*, p*_*yr*_*, p*_*zr*_] are the coordinates of the left and right pupil of the human eye in the camera coordinate system respectively. Therefore, it can be seen that the smaller ***R***_***g***_ is, the closer the gaze point is to the center of the facial plane of the robot, which implies that the more focused the gaze is on the robot and the more likely it is in the robot.

To indicate the impact factor of facial orientation features on HIE intuitively, first, we use the previously extracted features {***P***_***pitch***_, ***P***_***yaw***_, ***P***_***roll***_} to calculate the direction vector of face orientation from the 3D spatial rotation matrix transformation:

2 $${\varvec{V}}_{{\varvec{p}}} = \left[ {\begin{array}{*{20}c} { - cos{\varvec{P}}_{{{\varvec{pitch}}}} \cdot cos{\varvec{P}}_{{{\varvec{yaw}}}} } \\ { - sin{\varvec{P}}_{{{\varvec{pitch}}}} \cdot cos{\varvec{P}}_{{{\varvec{yaw}}}} } \\ {sin{\varvec{P}}_{{{\varvec{yaw}}}} } \\ \end{array} } \right]{ = }\left[ {\begin{array}{*{20}c} {v_{p1} } \\ {v_{p2} } \\ {v_{p3} } \\ \end{array} } \right]$$

Then, similar to ***R***_***g***_, ***R***_***p***_ is obtained by calculating the offset distance between the intersection of human face orientation and the center of robot face. The calculation formula is:

3 $${\varvec{R}}_{{\varvec{p}}} = \sqrt {(f_{x} - f_{z} \frac{{v_{p1} }}{{v_{p3} }})^{2} + (f_{y} - f_{z} \frac{{v_{p2} }}{{v_{p3} }})^{2} }$$

where (*f*_*x*_*, f*_*y*_*, f*_*z*_) is the central coordinate of human face in the camera coordinate system.

To reflect the features of HRI distance more intuitively and eliminate the influence of human height on interactive distance, we assume that both the robot head and human are in the same height plane, that is, ***T***_***py***_ = 0. Thus, the interactive distance between a human body and a robot can be calculated by formula (4).

4 $${\varvec{Dist}} = \sqrt {T_{px}^{2} + T_{pz}^{2} }$$

For the feature data of ***E***, we also hope to adopt a more effective and convenient method to analyze the correlation between expressions and human–robot engagement intentions. For this purpose, we refer to the method of Nurmi^36^ to divide the seven expression states into three categories: ‘Approach’, ‘Nature’ and ‘Avoid’. Then, we obtain the processed expression feature ***Exprs***, which is used to replace the feature data ***E***, as shown in Table 2. After the above processing, we can get four new features {***R***_***g***_, ***R***_***p***_, ***Dist****, ****Exprs***} with stronger readability.

**Table 2:** Classification of human expression according to the correlation between expressions and human–robot interactive intentions.

Expressions	***Exprs***
Happy	Approach
Expressionless	Nature
Surprised, sadness, disgusted, fear, angry	Avoid

#### Feature data test

So far, we have a total of 18-dimensional feature data that can be used to train our model. This set of features is denoted by X_18. We calculate the mean and standard deviation of the feature data for each dimension, and analyze the correlation between the feature data of each dimension ***x***_***i***_ and the sample label ***Y*** by using Pearson correlation coefficient^37^. The result is shown in Table 3. Among them, ***Exprs*** is the categorical feature which we use 1 (Avoid), 2 (Nature) and 3 (Approach) to represent the eigenvalues, respectively. We sorted all the features in descending order by the absolute value of the Pearson correlation coefficient |Pearson|. The larger the |Pearson|, the stronger the correlation between ***x***_***i***_ and ***Y***. It can be seen that the new features {***R***_***g***_, ***R***_***p***_, ***Dist****, ****Exprs***} are ranked at the top of Table 5. In addition, ***T***_***pz***_ and ***Dist*** are very similar because they both reflect distance information. The correlation between ***x***_***i***_ and ***Y*** has been obtained as an initial result, but it remains to be seen whether this relationship is consistent with the rules reflected in our machine learning model.

**Table 3:** Details of feature set data, including description, unit, mean, standard deviation and Pearson coefficient.

Symbol	Description	Unit	Mean	Std	Pearson
***R***_***g***_	Gaze intersection offset	mm	383.842	300.609	− 0.772
***R***_***p***_	Offset of face towards intersection	mm	721.055	761.970	− 0.668
***T***_***pz***_	Head translation variation z	mm	1043.253	302.724	− 0.628
***Dist***	Distance between the robot and the human	mm	1064.785	314.895	− 0.613
***Exprs***	Human facial expression	[1–3]	2.099	0.469	0.384
***V***_***gry***_	Right eye sight direction vector y	–	0.185	0.162	0.366
***G***_***pitch***_	Pitch angle of gaze	rad	0.186	0.169	0.335
***V***_***gly***_	Left eye sight direction vector y	–	0.167	0.159	0.333
***V***_***glx***_	Left eye sight direction vector x	–	0.054	0.243	0.214
***P***_***pitch***_	Pitch angle of head pose	rad	0.136	0.155	0.208
***G***_***yaw***_	Yaw angle of gaze	rad	− 0.031	0.255	0.191
***P***_***yaw***_	Yaw angle of head pose	rad	0.068	0.311	− 0.168
***V***_***grx***_	Right eye sight direction vector x	–	− 0.113	0.237	0.159
***T***_***px***_	Head translation variation x	mm	23.490	155.871	− 0.154
***T***_***py***_	Head translation variation y	mm	− 58.452	173.342	− 0.139
***V***_***grz***_	Right eye sight direction vector z	–	− 0.931	0.058	− 0.108
***V***_***glz***_	Left eye sight direction vector z	–	− 0.939	0.048	− 0.042
***P***_***roll***_	Roll angle of head pose	rad	− 0.112	0.145	− 0.021

### Engagement intention-intensity classifier

To predict the IHEI using multidimensional feature data, we adopt CatBoost, an advanced boosting ensemble learning model, to train the IHEI classifier. Based on oblivious trees, CatBoost enhances Gradient Boosting Decision Tree (GBDT)^38^ and can efficiently and reasonably handle categorical features.

The general processing method for categorical features is one-hot encoding, but this method might have the over-fitting problem. CatBoost uses a method based on the performance of randomization of data sets to process categorical features, and then computes the average target value of the sample based on the same category values placed before the randomization to reduce the occurrence of over-fitting. If we are given a dataset ${\text{D}}{ = }\{ (X_{i} ,y_{i} )\}_{n = 1,2,\ldots,n}$, where $X_{i} = (x_{i,1} ,...,x_{i,m} ) \in {\mathbb{R}}^{m}$ is a vector of m features (some numerical and some categorical),$Y_{i} \in {\mathbb{R}}$ is the label values. Call a set of permutations $\sigma = (\sigma_{1} ,...,\sigma_{n} )$, then $x_{{\sigma_{p,k} }}$ is replaced by:

5 $$\frac{{\sum\nolimits_{j = 1}^{p - 1} {[x_{{\sigma_{j,k} }} = x_{{\sigma_{p,k} }} ]} Y_{{\sigma_{j} }} + a \cdot P}}{{\sum\nolimits_{j = 1}^{p - 1} {[x_{{\sigma_{j,k} }} = x_{{\sigma_{p,k} }} ] + a} }}$$

Here, a prior value *P* and prior weight *a* (*a* > 0) are added to help reduce the noise obtained from low frequency classes.

### Model interpretability analysis

Although the machine learning model can effectively get the prediction results of HIE through the input feature data, the prediction model is a black box. We cannot find the execution principle behind the model intuitively. Another focus of our research in this paper is to do model interpretability analysis to study how the input features affect the HIE prediction model.

For some popular machine learning models, for instance, RF and CatBoost, traditional *feature_Importance_* function can only reflect the importance of features, but cannot reflect the specific influence of features on the prediction results. SHAP can provide a better scheme for explainable analysis of complex machine learning models and analysis of deeper connections and influences between features and models. The Shapley approach has the advantage of having strong theoretical support to ensure a fair attribution of features, thus fairly distributing the total predicted value in the features and their individual contributions.

SHAP uses game theory to calculate Shapley value and quantify the contribution each feature makes to model’s prediction results. The essential design idea of SHAP is:

- It first calculates the marginal contribution of a feature when it is added to the model.
- Then it calculates the different marginal contribution of the feature in all feature sequences.
- Finally, it calculates the Shapley value of the feature, that is, the mean value of all its marginal contributions.

### Ethics approval 

All of the authors confirm that there is no potential acts of misconduct in this work, and approve of the journal upholding the integrity of the scientific record.

### Consent to participate 

Informed consent was obtained from all individual participants included in the study.

## Experimental results and analysis

In this paper, the IHEI recognition was carried out in an indoor environment, with a certain distance as the prerequisite to ensure effective interactive communication activities between people. Proxemics^9^ divided interactive distance into four categories: intimate distance (0–45 cm), personal distance (45–120 cm), courtesy distance (120–360 cm), and general distance (more than 360 cm). For the social communication purpose, we targeted the human intention within the range of 0–360 cm.

### Data set acquisition

14 participants from different majors in Chongqing University of Technology were recruited to participate in the study (age range 18–25 years old, M_age_ = 21.07, 8 males). None of them had previously participated in similar human–robot interaction experiments, and none had been exposed to our social robot. We told participants in advance about the capabilities of our social robot and the reliability of the technology involved. All of them had a high level of trust in our technology.

The experiment area we chose was in a public room. We designated a certain area of the room for our experiment. Before entering the room, outsiders will be prompted not to enter the experimental area without permission. In this area, in addition to our social robot, there were other potentially distracting devices around it, such as books on the table, and LCD display on the wall. In addition, the interaction function of our social robot is relatively limited, and it can only carry out simple speech conversations (greet, introduce some information about itself) and make simple expressions (smile). Participants can experience all the functions of the robot in a short time (about 2 min).

**Figure 4:**
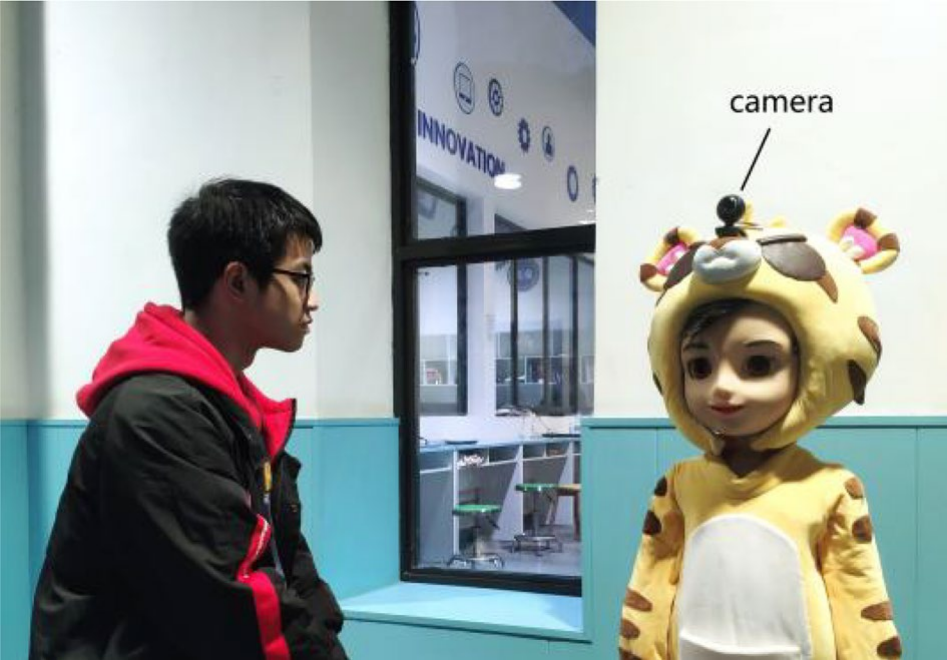
Participants were interacting with the social robot. The robot, wearing yellow clothes on the right, has a camera placed above its head to capture the interaction data of participants.

We arranged one participant at a time to enter the experiment area to interact with the robot as shown in Fig. 4. When the participant enters the experiment area, a 30-FPS monocular camera is placed by the robot’s head to continuously extract the participant’s visual features, and the feature data together with the video are saved locally. Before the experiment, all the participants were informed of the same information as follows: (1) we have placed our social robot that can perform a simple speech conversation and make simple expressions in the experiment area. (2) After the participant enters the experiment area, the robot needs to be prepared for 2 min to start the interaction and he can familiarize yourself with the environment during the waiting period. (3) After 2 min (the robot will give a voice prompt), the participant can choose to interact with the robot, or can choose not to interact with the robot and do other things he like to do, such as reading a book, listening to music and so on. (4) Each one should stay in the experiment area for at least 5 min.

**Figure 5:**
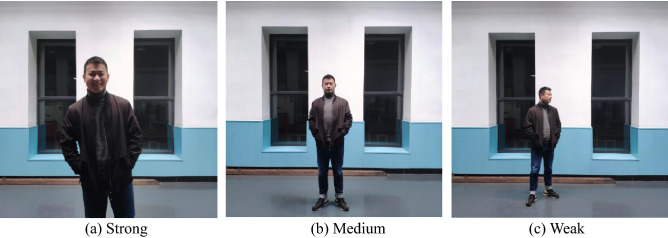
Hierarchy of IHEI among participants. The IHEI is divided into three levels: strong, medium and weak.

After the experiment, we informed participants about the specifics of the experiment we conducted and explained to them the concept of IHEI. Finally, we can get 14 sets of data, each of which is filtered to get 6000 frames of high-quality feature data. Within each set of data, we split the saved video into pictures by frame and matched the 6000 frames of data mentioned above with the corresponding picture. Further, participants watched the interaction video and self-reported the IHEI for the interaction in 6000 pictures, that is, a classification label for these 6000 frame feature data. To reduce the variability of self-assessment, the IHEI was classified as strong, medium and weak, as shown in Fig. 5. Among them, ‘Strong’ means that the person is currently very interested in interacting with the robot. ‘Weak’ means that the person hardly wants to interact with the robot, while ‘Medium’ is between ‘Strong’ and ‘Weak’, which means that the person is only interested in the robot rather than interacting with it. These three levels were reviewed and verified by experts.

**Figure 6:**
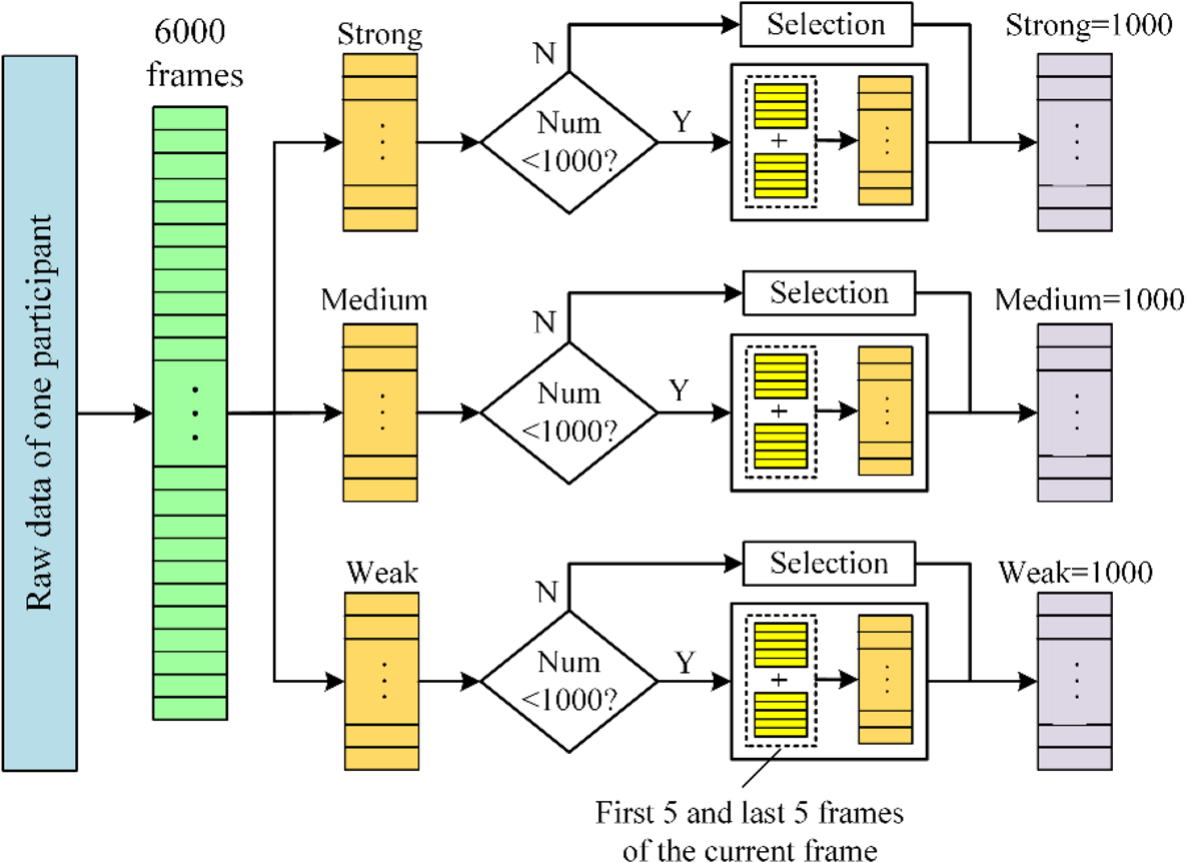
Method to balancing three categories of data in our dataset.

Furthermore, we used the method shown in Fig. 6 to selected 3000 frames of data from 6000 frames of data, with 1000 frames of intensity for each of the three types of IHEI, to ensure uniform distribution of sample sets from different categories. As can be seen from Fig. 6, for the IHEI category with less than 1000 frames, we randomly selected 5 frames of valid data before and after the current frame to add to the current category data, and the label is the same as the current category, until the total number of the category reaches 1000 frames. The new frames of data do not contain the existing data of the current category. Our feature extraction algorithm can reach 30FPS, so the data difference between 5 frames before and after the current frame will be small. The addition of new data can effectively expand the category of data with insufficient total number. Thus, an IHEI recognition dataset containing 42,000 samples can be obtained.

In addition, the reasons why only one participant is selected each time instead of several participants interacting with the robot are as follows: (1) we can get higher quality data sets by extracting face features of a single person each time and facilitating subsequent processing; (2) the final trained IHEI classifier is applicable to everyone, because each person has the same feature input and the engagement behavior of each person under different IHEI is overall similar, so it can be used for multi-person detection. (3) We assume that people determine that individuals’ IHEIs to social robots are unaffected by others in the present environment, i.e., judgments regarding an individual’s IHEIs to the social robot are independent. (4) We expect machine learning model to learn general trends and laws while accommodating individual variability. Then, we can use less resources to get a more applicable model to analyze people's IHEI.

### Classifier training and evaluation

#### Split of dataset

Of these 14 sets of data, ten sets were used as training sets for the IHEI classifier, denoted by D_10, and the remaining four sets were used for testing, denoted by D_4. This division is done to avoid the fact that data from the same individual are used for both training and testing, thus reducing the generalization ability of the model. In the process of training the IHEI classifier, we need to divide some data from the training set as a validation set to determine the optimal number of model iterations. In order to make full use of all the data in the training set, K fold cross validation^39^ is used as a strategy to divide the training set and the validation set during model training. K fold cross validation avoids the extremes of uneven distribution of the training and validation sets due to differences in different batches of characterization data.

#### Classifier evaluation

In order to verify the effectiveness of CatBoost, we also evaluated the other machine learning models commonly used for intention classification, including SVM, RF and MLP using the same dataset (D_4). Among them, SVM is generally used to deal with binary classification problems. In the case of multi-classification problems, the multi-classification problems can be converted into binary classification problems by using one-to-rest mode. In addition, since SVM, RF and MLP cannot train the category features directly, we use one-hot coding to preprocess ***Exprs*** features before the training.

We choose the weighted-F1 score and test time as the model evaluation indexes in the test set to evaluate the SVM, RF and MLP models, respectively. The weighted-F1 score is chosen to evaluate the overall predictive performance of the model, which was calculated by Eq. (6), where *P*_*i*_ and *R*_*i*_ are the precision and recall of the prediction results of each type of label, respectively, *w*_*i*_ is the ratio of the number of samples in each category to the total number of samples.

6 $$weighted{ \text{-} }F{1} = \frac{{2 \times \sum\limits_{i = 1}^{n} {w_{i} P_{i} } \times \sum\limits_{i = 1}^{n} {w_{i} R_{i} } }}{{\sum\limits_{i = 1}^{n} {w_{i} P_{i} } + \sum\limits_{i = 1}^{n} {w_{i} R_{i} } }}$$

In addition, in order to verify that the intent classification model trained by the optimized data set has better performance, three data set combinations are obtained by combining features of different dimensions, as shown in Table 4. X_15 represents the combination of original 15-dimensional feature data extracted, X_4 represents the combination of four advanced features obtained through data enhancement. Subsequently, SVM, RF, MLP and CatBoost were trained and tested on these three datasets, respectively. The final evaluation results are shown in Table 5.

**Table 4:** Three combinations of datasets.

Dataset	Feature set
X_15	15-dimensional raw feature data
X_4	***R***_***g***_, ***R***_***p***_, ***Dist****, ****Exprs***
X_18	18-dimensional feature data

**Table 5:** Performance comparison of three classifiers in three datasets.

Dataset	Classifier	Weighted-F1 score	Time (s)
X_15	SVM	0.4519	2.1667
	MLP	0.4426	0.0407
	RF	0.6657	0.0591
	CatBoost	0.6968	0.0179
X_4	SVM	0.9008	0.2313
	MLP	0.8829	0.0450
	RF	0.8980	0.0523
	CatBoost	0.9027	0.0124
X_18	SVM	0.5106	0.1572
	MLP	0.8017	0.0413
	RF	0.9029	0.0518
	CatBoost	0.9112	0.0131

It can be seen that the test performance of the four classifiers on X_18 and X_4 dataset is generally superior to X_15 datasets, which indicates that the four high-level features we added are beneficial to the training of the classifier. Meanwhile, among all the classifiers, CatBoost has the highest weighted-F1 score and the lowest test time, indicating that its overall test performance is better than other classifiers. This is also in accordance with our expected conjecture.

### SHAP analysis

In order to further explore the deep relationship between features and the IHEI, the SHAP model is used to perform interpretability analysis on the trained CatBoost-based classifier for the IHEI. Based on calculated results, the influencing factors of IHEI classification are analyzed from both global and individual aspects.

#### Global analysis

In global analysis, the average absolute value of each characteristic SHAP value is used to measure its global importance. At the same time, by analyzing the distribution relationship between the value of each feature and its SHAP value, we can get both a positive and a negative correlation between the feature and the evaluation results.

**Figure 7:**
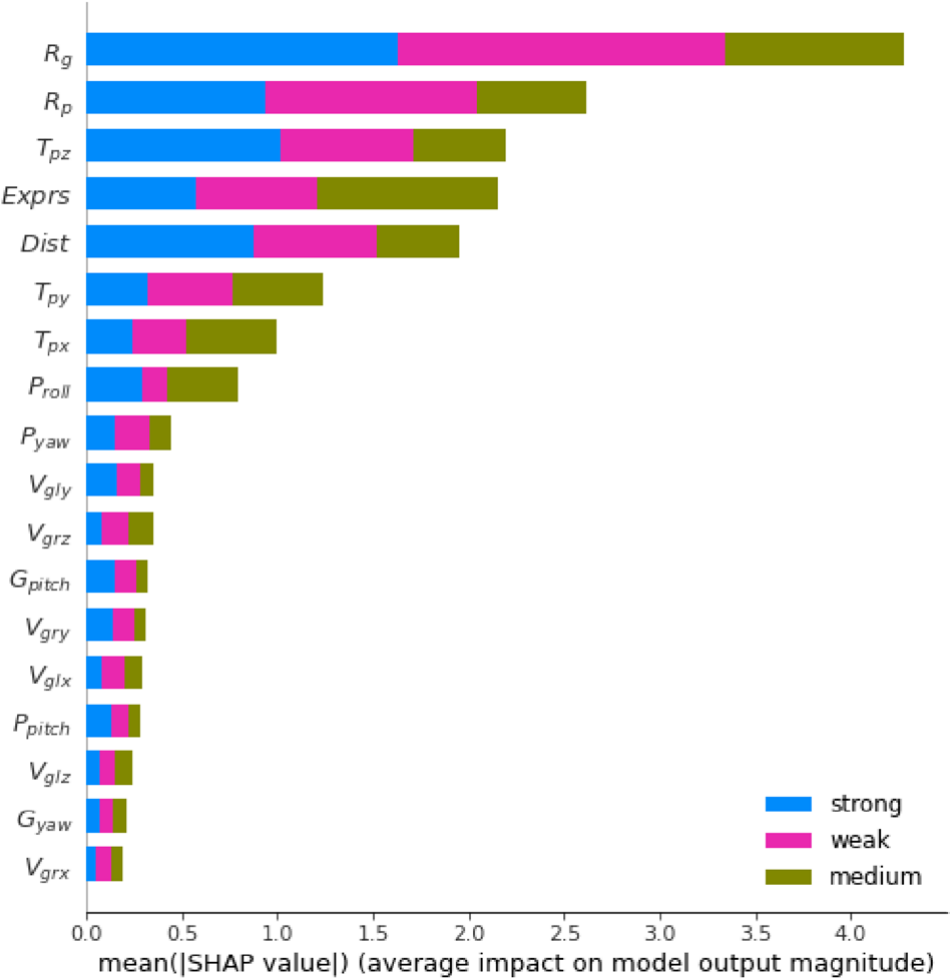
The effects of each feature on the intensity of different IHEI (strong, medium and weak) are obtained by the SHAP analysis. The importance of each feature is ranked in descending order.

First, the influence of various features on different labels (IHEI) is analyzed by using the SHAP model, as shown in Fig. 7, where the horizontal axis represents the mean (|SHAP value|) of various features, the vertical axis represents 18 types of features, and three different color blocks represent three types of labels.

Among the top five features, there are four readability enhancement features, i.e., ***R***_***g***_*,**** R***_***p***_*, ****Dist****,* and ***Exprs***, to which, in comparison with the original features, the IHEI is more sensitive. Their order of importance is ***R***_***g***_ > ***R***_***p***_ > ***Exprs*** > ***Dist***. This implies that the features reflecting human attention play a dominant role in the classification of the IHEI.

**Figure 8:**
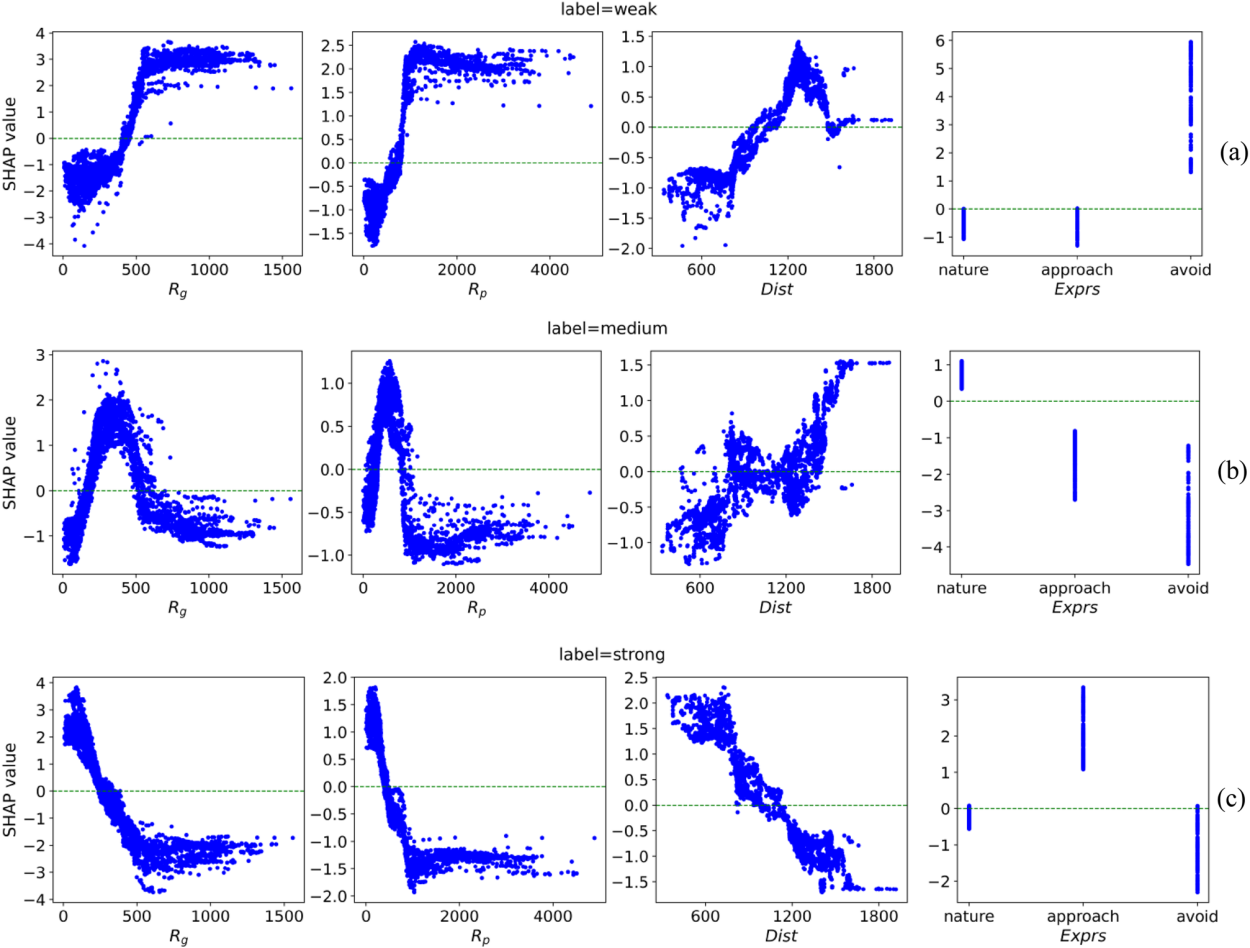
Distribution of the shape values of ***R***_***g***_, ***R***_***p***_, ***Dist*** and ***Exprs*** under different IHEI labels (i.e., strong, medium, and weak). (**a**) Weak HIE intensity. (**b**) Medium HIE intensity. (**c**) Strong HIE intensity.

Second, we analyzed the distribution of the SHAP values for the four features processed by readability enhancement relative to different IHEI labels, as shown in Fig. 8. Among them, a SHAP value greater than 0 indicates that the current feature value has a positive promoting effect on this IHEI, and a larger SHAP value indicates that the current feature can reflect a stronger IHEI. SHAP values less than 0 indicates inverse contributions to the current eigenvalue, and the smaller the SHAP value, the greater the inhibition.

- label = Weak

In this state, the SHAP values of ***R***_***g***_, ***R***_***p***_ and ***Dist*** are approximately inversely proportional to the feature data. The larger their values are, the stronger the promotion of weak HIE is. In addition, the feature values at SHAP turning points (SHAP value is 0) of ***R***_***g***_, ***R***_***p***_ and ***Dist*** are approximately 440 mm, 750 mm, and 1010 mm, respectively. For ***Exprs***, only when the expression category is ‘Avoid’, there is a significant promotion effect on weak HIE.

(b) label = Medium

For ***R***_***g***_, ***R***_***p***_ and ***Dist***, the relationship between their SHAP values and the trait data is relatively complex and cannot be analyzed with a simple linear description. From the distribution of the SHAP values of ***Exprs***, it can be observed that ‘Nature’ expression category has an intuitive effect on such IHEI.

(c) label = Strong

It can be seen that the distribution of SHAP values of various features is approximately the opposite to that when the label is Weak, the SHAP values of ***R***_***g***_, ***R***_***p***_ and ***Dist*** are approximately proportional to the feature data. Meanwhile, the feature values at the SHAP turning point (SHAP value is 0) of ***R***_***g***_, ***R***_***p***_ and ***Dist*** are approximately 300 mm, 500 mm and 1,050 mm, respectively. In addition, at this time, the expression type of ‘Approach’ plays a role in promoting the HIE intensity state.

According to the distribution of SHAP values of these four types of features, we sort out the areas where they play a positive role in promoting various labels, as shown in Table 6. It can be found that although SHAP values with different features show certain rules for the change of IHEI, such rules cannot be perfectly interpreted by a few rules, because some dependencies are relatively complex, such as distance data with medium HIE intensity. In addition, we can see that the distribution results of the SHAP value of ***Dist*** are related to proxemics, but there are slight differences. When IHEI is strong, it is basically within intimate distance and personal distance; when IHEI is weak, it is considered outside of the personal distance. However, when IHEI is medium, the situation is more complicated, so it is not complete to judge IHEI from spatial distance.

**Table 6:** The change rule of eigenvalue and intensity of interactive intention.

Feature	Weak	Medium	Strong
***R***_***g***_ (mm)	> 440	(180, 550)	< 300
***R***_***p***_ (mm)	> 750	(280, 900)	< 500
***Dist*** (mm)	> 1010	–	< 1050
***Exprs***	Avoid	Nature	Approach

The analyzing results show that although rule-based intent recognition is efficient, it has a low error tolerance rate, and it is difficult to deal with problems in particular circumstances. Undoubtedly, the above results provide a relevant base for HRI decision-making, as well as a means for non-professionals to develop HRI applications.

#### Individual analysis

**Figure 9:**
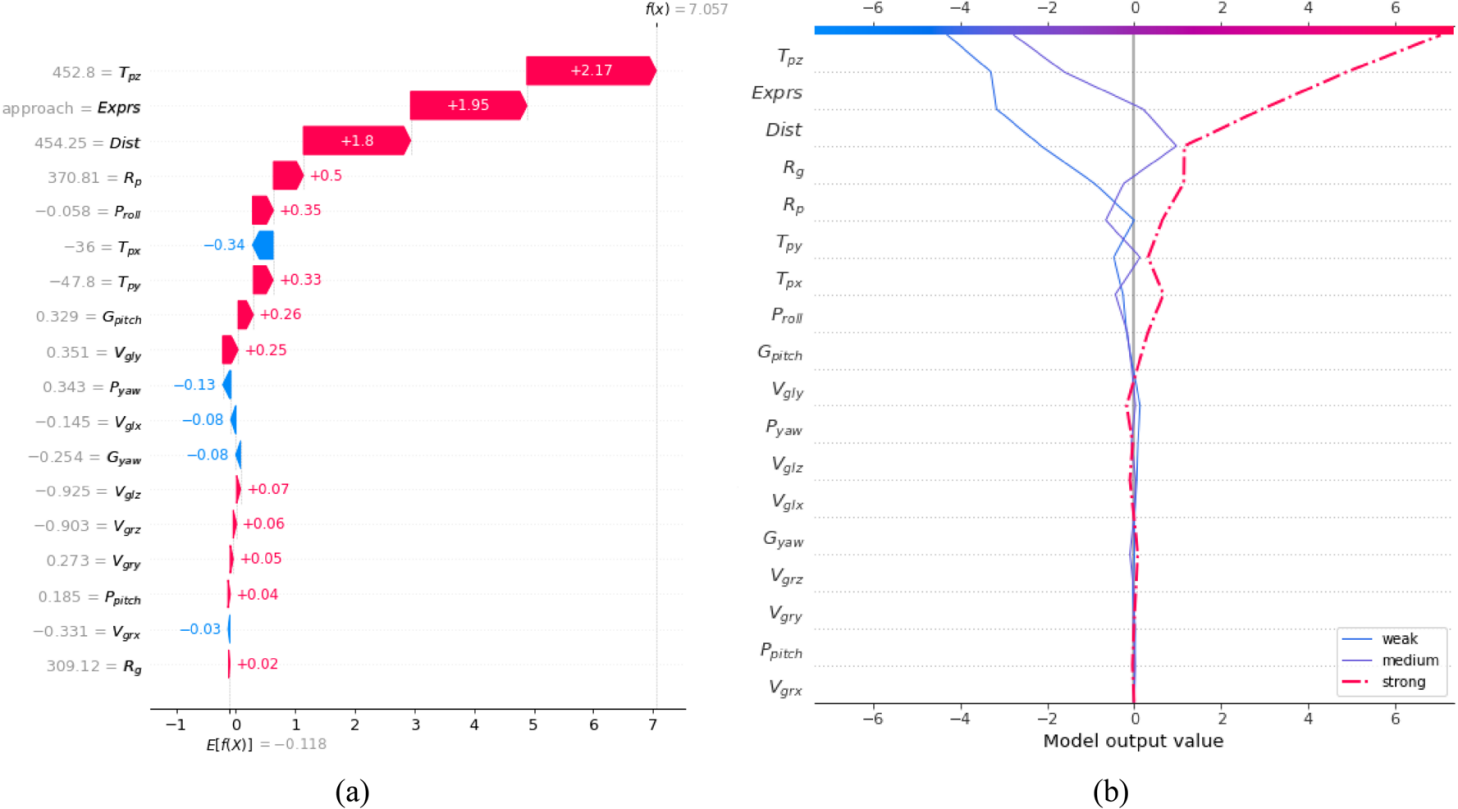
SHAP analysis of an example where the IHEI is predicted to be strong. (**a**) The degree to which each feature contributes to the predicted result of the interactive intention (corresponding value shown on the right side of each feature). Red indicates that the feature has a positive effect on the predicted result, whereas blue indicates that the feature has a negative effect. (**b**) The influence of each feature on the trend of different prediction results. Prediction results are presented based on the category of interactive intention that has the highest SHAP value.

In the individual analysis, we are able to understand the key factors affecting this sample status level by calculating the SHAP values for all the features for a single sample, and in turn, to make clear which special feature values are responsible for this effect. Figure 9 shows an example where the IHEI is predicted to be strong, including the contribution of a single feature to the IHEI.

In Fig. 9a, the red colors indicate that the SHAP value is positive, which will increase the predicted value in the evaluation process, while the blue ones show the contrast. The baseline value of the model *E*[*f(x)*] is − 0.118, and the final evaluation of the strength of interactive *f*(*x*) *i*s obtained by adding the SHAP value of each feature to the baseline value. It can be seen that the SHAP values of ***Tpz***, ***Exprs*** and ***Dist*** are positive and large, which play a significant role in predicting the IHEI in this sample.

Figure 9b illustrates the predicted trend of different labels. The intersection between the top of the curve and the coordinate axis is the SHAP value of the IHEI. It can be seen that only the SHAP value for which the prediction result is strong is positive, i.e., the sample has the highest probability of being predicted as strong HIE. Meanwhile, ***Tpz***, ***Exprs*** and ***Dist*** have the greatest influence on the trend of the three predicted curves. It can be seen that in this example, the distance feature has the greatest influence on the prediction of IHEI.

## Discussion

As the advanced integrated learning model proposed at present, CatBoost can effectively train the interactive intent dataset we have built, and it shows better performance than other models we chose. The IHEI classifier trained by CatBoost has a weighted-F1 value of 0.9112 on the test set. This indicates that the classifier is able to effectively predict three intensities of HIE.

Since it is a preliminary exploration of the relationship between visual features and the IHEI, we have only divided into three types of IHEIs. However, the situation in real life might be more complex, and in real-time engagement intention recognition for multiple people, the recognition results need to be further refined to effectively distinguish different people. A future research direction will be to classify the IHEI into more detailed levels. In addition, we assume that the IHEI of a robot is absolute, not relative, and by default unaffected by other people. We will conduct experiments in the future to examine how the relative IHEI between people affects the assessment of the final IHEI.

Through the interpretability analysis based on the classification model by SHAP, the importance ranking of all types of feature data can be preliminarily achieved, with ***R***_***g***_ and ***R***_***p***_ ranking as the first two in the data associated with the line of sight and face orientation, respectively. Both ***R***_***g***_ and ***R***_***p***_ reflect the current state of human attention, that is, the area of interest. This shows that, compared with other features mentioned in this paper, attention information can best reflect the state of IHEI.

SHAP analysis provides an effective basis for our real-time IHEI recognition link. Following this research, we propose to build a real-time IHEI recognition model based on the analysis obtained in the Section of SHAP analysis. Using this model, robots are able to achieve more natural HRI by selecting a reasonable interaction target when they interacting with multiple people.

## Conclusion

It is of great importance for the study of natural HRI to enable social robots to perceive the IHEI. In this paper, a deep analysis of IHEI is carried out using a CatBoost-based model and SHAP. During feature engineering, four types of high level features related to human line of sight, head posture, distance, and facial expressions can be obtained. A dataset of IHEI is created by categorizing IHEIs into strong, medium, and weak, according to their intensity. Using the obtained dataset, a CatBoost-based IHEI classification model is then trained. By applying the SHAP, an interpretability analysis is conducted for the trained classification model, with a view to exploring the deep relationship between the features set and IHEI. This is conducive to human behavior research and allows non-professionals to develop HRI more efficiently.

## Data Availability

The datasets generated during the current study are available from the corresponding author on reasonable request.

## References

[1] Xue, Y., *et al*. Proactive interaction framework for intelligent social receptionist robots. In *2021 IEEE International Conference on Robotics and Automation (ICRA), May 30–June 5, 2021, Xi'an, China*, pp. 3403–3409. IEEE. 10.1109/ICRA48506.2021.9562115.

[2] Salichs MA, Castro-González Á, Salichs E (2020). Mini: A new social robot for the elderly. Int. J. Soc. Robot..

[3] Chen H, Park HW, Breazeal C (2020). Teaching and learning with children: Impact of reciprocal peer learning with a social robot on children’s learning and emotive engagement. Comput. Educ..

[4] Ramanathan, M., Mishra, N., & Thalmann, N. M. Nadine humanoid social robotics platform. In *Computer Graphics International Conference, June 17-June 20, 2019, Calgary, Canada*, 490–496 (Springer). 10.1007/978-3-030-22514-8_49.

[5] Heenan, B., Greenberg, S., *et al*. Designing social greetings in human robot interaction. In *Proceedings of the 2014 conference on Designing interactive systems, June 21–25, 2014, New York, United States*, pp. 855–864. 10.1145/2598510.2598513.

[6] Michalowski, M. P., Sabanovic, S., & Simmons, R. A spatial model of engagement for a social robot. In *9th IEEE International Workshop on Advanced Motion Control, March 27–29, 2006, Istanbul, Turkey*, pp. 762–767. IEEE. 10.1109/AMC.2006.1631755.

[7] Feil-Seifer D, Matarić MJ (2012). Distance-based computational models for facilitating robot interaction with children. J. Human-Robot Interact..

[8] Bi, J., *et al*. Interactive intention prediction model for humanoid robot based on visual features. In *2nd International Conference on Control, Robotics and Intelligent System**, **August 20–22, 2021, Qingdao, China*, pp. 36–41. 10.1145/3483845.3483852.

[9] Hall ET, Birdwhistell RL, Bock B (1968). Proxemics [and comments and replies]. Curr. Anthropol..

[10] Walters, M. L., *et al*. An empirical framework for human-robot proxemics. In *Proceedings of New Frontiers in Human–Robot Interaction* 2009.

[11] Mumm, J., & Mutlu, B. Human-robot proxemics: Physical and psychological distancing in human-robot interaction. In *Proceedings of the 6th International Conference on Human–Robot Interaction, March 6–9, 2011, Lausanne, Switzerland*, pp. 331–338. 10.1145/1957656.1957786.

[12] Zhao, Q., *et al*. Natural human-robot interaction for elderly and disabled healthcare application. In *IEEE International Conference on Bioinformatics and Biomedicine (BIBM), November 2–5, 2014, Belfast, United Kingdom*, pp. 39–44. IEEE. 10.1109/BIBM.2014.6999239.

[13] Kobayashi, Y., *et al*. A considerate care robot able to serve in multi-party settings. In *20th IEEE International Symposium on Robot and Human Interactive Communication (RO-MAN), July 31-August 3 ,2011, Atlanta, Georgia*, pp. 27–32. IEEE. 10.1109/ROMAN.2011.6005286.

[14] Ozaki, Y., *et al*. Decision-making prediction for human-robot engagement between pedestrian and robot receptionist. In *27th IEEE International Symposium on Robot and Human Interactive Communication (RO-MAN), August 27–31, 2018, Nanjing, China*, pp. 208–215. IEEE. 10.1109/ROMAN.2018.8525814.

[15] Mazhar, O., *et al*. Towards real-time physical human-robot interaction using skeleton information and hand gestures. In *IEEE/RSJ International Conference on Intelligent Robots and Systems (IROS), October 1–5, 2018, Madrid, Spain*, pp. 1–6. IEEE. 10.1109/IROS.2018.8594385.

[16] Koo, S., & Kwon, D. S. Recognizing human intentional actions from the relative movements between human and robot. In: 18th IEEE International Symposium on Robot and Human Interactive Communication, September 27–October 2, 2009, Toyama, Japan, pp. 939–944. IEEE. 10.1109/ROMAN.2009.5326127.

[17] Kelley, R., *et al*. Understanding human intentions via hidden Markov models in autonomous mobile robots. In *Proceedings of the 3rd ACM/IEEE international conference on Human robot interaction (HRI), March 12–15, 2008, Amsterdam, Netherlands*, pp. 367–374. 10.1145/1349822.1349870.

[18] Kato, Y., Kanda, T., & Ishiguro, H. May i help you?-design of human-like polite approaching behavior. In *10th ACM/IEEE International Conference on Human–Robot Interaction (HRI), March 2–5, 2015, Portland*, pp. 35–42. IEEE.

[19] Cortes C, Vapnik V (1995). Support-vector networks. Mach. Learn..

[20] Vaufreydaz D, Johal W, Combe C (2016). Starting engagement detection towards a companion robot using multimodal features. Robot. Auton. Syst..

[21] Sidiropoulos, G. K., *et al*. Measuring engagement level in child–robot interaction using machine learning based data analysis. In *International Conference on Data Analytics for Business and Industry: Way Towards a Sustainable Economy, October 26–27, 2020, Sakheer, Bahrain*, pp. 1–5. IEEE. 10.1109/ICDABI51230.2020.9325676.

[22] Seber GA, Lee AJ (2012). Linear Regression Analysis.

[23] Hornik K, Stinchcombe M, White H (1989). Multilayer feedforward networks are universal approximators. Neural Netw..

[24] Breiman L (2001). Random forests. Mach. Learn..

[25] Prokhorenkova L, Gusev G (2018). CatBoost: Unbiased boosting with categorical features. Adv. Neural Inf. Process. Syst..

[26] Dorogush, A. V., Ershov, V., & Gulin, A. CatBoost: Gradient boosting with categorical features support. arXiv:1810.11363 (arXiv preprint) (2018).

[27] Lundberg, S. M., & Lee, S. I. A unified approach to interpreting model predictions. In *Proceedings of the 31st international conference on neural information processing systems**, **December 4–9, 2017, California, USA*, pp. 4768–4777.

[28] Baltrusaitis, T., *et al*. OpenFace 2.0: Facial behavior analysis toolkit. In *13th IEEE International Conference on Automatic Face and Gesture Recognition, May 15–19, 2018, Xi'an, China*, pp. 59–66. IEEE. 10.1109/FG.2018.00019.

[29] Baltrusaitis, T., Robinson, P., & Morency, L. P. Constrained local neural fields for robust facial landmark detection in the wild. In *Proceedings of the IEEE International Conference on Computer Vision Workshops, December 2–8, 2013, Sydney, Australia*, pp. 354–361. IEEE. 10.1109/ICCVW.2013.54.

[30] Wood, E., *et al*. Rendering of eyes for eye-shape registration and gaze estimation. In *Proceedings of the IEEE International Conference on Computer Vision Workshops, December 7–13, 2015, Santiago, Chile*, pp. 3756–3764. IEEE. 10.1109/ICCV.2015.428.

[31] Zadeh, A., *et al*. Convolutional experts constrained local model for 3d facial landmark detection. In *Proceedings of the IEEE International Conference on Computer Vision Workshops, October 22–29, 2017, Venice, Italy*, pp. 2519–2528. IEEE. 10.1109/ICCVW.2017.296.

[32] Fiore SM, Wiltshire TJ, Lobato EJ (2013). Toward understanding social cues and signals in human-robot interaction: Effects of robot gaze and proxemic behavior. Front. Psychol..

[33] Truong, X. T., & Ngo, T. D. Social interactive intention prediction and categorization. In *ICRA 2019 Workshop on MoRobAE-Mobile Robot Assistants for the Elderly, May 20–24, 2019, Montreal, Canada*.

[34] Ekman P, Rosenberg EL (1997). What the Face Reveals: Basic and Applied Studies of Spontaneous Expression Using the Facial Action Coding System (FACS).

[35] Tian YI, Kanade T, Cohn JF (2001). Recognizing action units for facial expression analysis. IEEE Trans. Pattern Anal. Mach. Intell..

[36] Nurmi JE, Toivonen S, Salmela-Aro K (1996). Optimistic, approach-oriented, and avoidance strategies in social situations: Three studies on loneliness and peer relationships. Eur. J. Pers..

[37] Benesty J, Chen J, Huang Y (2009). Pearson correlation coefficient. Noise Reduction in Speech Processing.

[38] Friedman JH (2001). Greedy function approximation: A gradient boosting machine. Ann. Stat..

[39] Bengio Y, Grandvalet Y (2004). No unbiased estimator of the variance of k-fold cross-validation. J. Mach. Learn. Res..

